# Multi-tissue transcriptome analysis using hybrid-sequencing reveals potential genes and biological pathways associated with azadirachtin A biosynthesis in neem (*azadirachta indica*)

**DOI:** 10.1186/s12864-020-07124-6

**Published:** 2020-10-28

**Authors:** Huiyan Wang, Ning Wang, Yixin Huo

**Affiliations:** 1 Key Laboratory of Molecular Medicine and Biotherapy, School of Life Science, Beijing Institute of Technology, No. 5 South Zhongguancun Street, Beijing, 100081 P.R. China; 2 SIP-UCLA Institute for Technology Advancement, 10 Yueliangwan Road, Suzhou Industrial Park, Suzhou, 215123 P.R. China

**Keywords:** Azadirachtin A, Natural insecticides, Secondary metabolism, Triterpenoid biosynthesis, Transcriptome, Neem

## Abstract

**Background:**

Azadirachtin A is a triterpenoid from neem tree exhibiting excellent activities against over 600 insect species in agriculture. The production of azadirachtin A depends on extraction from neem tissues, which is not an eco-friendly and sustainable process. The low yield and discontinuous supply of azadirachtin A impedes further applications. The biosynthetic pathway of azadirachtin A is still unknown and is the focus of our study.

**Results:**

We attempted to explore azadirachtin A biosynthetic pathway and identified the key genes involved by analyzing transcriptome data from five neem tissues through the hybrid-sequencing (Illumina HiSeq and Pacific Biosciences Single Molecule Real-Time (SMRT)) approach. Candidates were first screened by comparing the expression levels between the five tissues. After phylogenetic analysis, domain prediction, and molecular docking studies, 22 candidates encoding 2,3-oxidosqualene cyclase (OSC), alcohol dehydrogenase, cytochrome P450 (CYP450), acyltransferase, and esterase were proposed to be potential genes involved in azadirachtin A biosynthesis. Among them, two unigenes encoding homologs of MaOSC1 and MaCYP71CD2 were identified. A unigene encoding the complete homolog of MaCYP71BQ5 was reported. Accuracy of the assembly was verified by quantitative real-time PCR (qRT-PCR) and full-length PCR cloning.

**Conclusions:**

By integrating and analyzing transcriptome data from hybrid-seq technology, 22 differentially expressed genes (DEGs) were finally selected as candidates involved in azadirachtin A pathway. The obtained reliable and accurate sequencing data provided important novel information for understanding neem genome. Our data shed new light on understanding the biosynthesis of other triterpenoids in neem trees and provides a reference for exploring other valuable natural product biosynthesis in plants.

## Background

With the increasing concern on the threat of chemical pesticides to global crop protection programs, more attention is being paid towards bioactive and biodegradable plant or microbial-based biopesticides. Azadirachtin A, the major insecticidal ingredient in neem-based products, exhibits excellent bioactivity against over 600 insect species [1] in agricultural areas [2]. It is processed by insects as a natural hormone and it induces antifeedant, repellent, and growth inhibiting behavior in insects [3]. Azadirachtin A-based pesticides are biodegradable, environment-friendly, and non-toxic to mammals, plants, and birds. Due to these general superior characteristics of azadirachtin A, the agriculture segment accounts for the highest share (40%) in total revenues of neem extract product market. This market is expected to grow from $653 million in 2015 to $1.8 billion in 2022 with a high annual growth rate of 16.3% [4]. The current supply of azadirachtin A mostly depends on extracts from neem seeds [5] and leaves. Due to the limitation of the distribution of neem materials along with the complex and low-yield azadirachtin A-extracting approach, the production of azadirachtin A is far from meeting the market demand.

Being a potential insecticide, the synthesis of azadirachtin has been investigated over the last few decades. However, the complexity in its molecular architecture [6] was the main obstacle that held back the advances in azadirachtin A biosynthesis. After 20 years of investigation, the complete chemical synthesis of azadirachtin A was finally accomplished [7] in 2007. However, the low productivity (0.00015%) of the 71 step-reaction still limits its applications in the industry.

Being a triterpenoid from neem, azadirachtin A is derived from 2,3-oxidosqualene. The downstream biosynthesis pathway, that is, the pathway from the biosynthesis of scaffold to azadirachtin A remains unclear after decades of investigation. One of the attempts at the investigation of its biosynthesis was the feeding experiments in 1971, [^3^H] euphol and [^3^H] tirucallol were incubated with neem leaf crude extract. Euphol, rather than tirucallol was more efficiently incorporated into nimbolide (a limonoid with structural similarity to azadirachtin A) [8]. However, no enzyme producing tirucallol or euphol was isolated or characterized from neem.

Several resources on neem genome, such as the complementary DNA (cDNA) library [9], expressed sequence tag library [10], draft genome [11, 12] and transcriptome data [13–15] are available in public databases such as the National Center for Biotechnology Information (NCBI) and the European Bioinformatics Institute (EBI). Genes involved in the mevalonate (MVA) pathway, methylerythritol phosphate (MEP) pathway [14, 16], or 2,3-oxidosquanlene biosynthesis [11, 15–17] in neem have been identified. Several neem-specific genes were found after comparative genome analysis of neem with *Arabidopsis thaliana*, *Oryza sativa,* and *Citrus sinensis* [11]. Upon comparison of gene expression within different neem tissues, genes encoding cytochromes P450 (CYP450s) were identified from fruit [16] and leaf [17]. However, none of the isolated genes had been functionally characterized. It was not until the key enzyme that converts 2,3-oxidosqualene into tirucalla-7,24-dien-3β-ol, AiOSC1, was isolated from neem transcriptome [18]. This was the first functionally characterized AiOSC1 reported to be related to azadirachtin scaffold biosynthesis. Two full-length CYP450s subsequently catalyzing tirucalla-7,24-dien-3β-ol into melianol were also isolated from *C. sinensis* var. Valencia and *Melia azedarach*. The homolog of MaCYP71CD2 in neem (AiCYP71CD2) was identified, the homolog of MaCYP71BQ5 (a characterized CYP450 from *M. azedarach*) [18] in neem was fragmented.

Although the genome and transcriptome data were easy to download from the Sequence Read Archive (SRA) database, the submitted files included all raw data without genome assembly or annotation. Besides, samples used in the previous reports [11, 12, 14–16] were from India. Genetic information within the same species found in different areas or developmental stages were different, this caused differences in gene expression. Hence, five neem tissues (fruit, leaf, stem, flower, and root) were sampled for transcriptome sequencing. Among the five tissues, fruit with the green hard seed has been reported to contain the highest amount of azadirachtin A throughout fruit development [14, 19] Leaf sample from a neem tree in China contained azadirachtin A at a concentration of 969.9 μg/g [20]. The percentage azadirachtin content in different tissues was consistent with that in a previous report (seed kernels, 0.03%; leaves, 0.9 × 10^− 3^%; bark, 0.5 × 10^− 3^%; root, 0.3 × 10^− 3^%; stem 0.2 × 10^− 3^%) [21]. Hence, these samples were chosen as the higher azadirachtin A group and used for mining genes for azadirachtin A biosynthesis.

With the advances in high-throughput sequencing technologies, the third-generation sequencing represented by PacBio Single Molecule Real-Time (SMRT) technology [22] and Oxford Nanopore sequencing has been applied in academic research. Due to the high error rate of sequencing longer reads (15%) in third-generation sequencing as well as the accuracy of reads from Illumina sequencing, a new method called hybrid-seq [23] has been generated, that brings together the best of two sequencing technologies. The longer reads obtained are corrected by short but accurate reads from Illumina. As reported by Koren [24], only 0.1% (41) of the PacBio reads exactly matched the annotated exon structure before correction during genome assembly. This percentage will rise to 24.1% (12,065) after correction with short reads. Error correction method was used for de novo genome assembly of *Saccharomyces cerevisiae*. The generated contig N50 length is more than ten times greater than an Illumina-only assembly (678 kb versus 59.9 kb) and has > 99.88% consensus identity when compared to the reference genome [25]. The successful applications of hybrid-seq in genome refining and isoform identification laid strong foundations for our study.

**Fig. 1:**
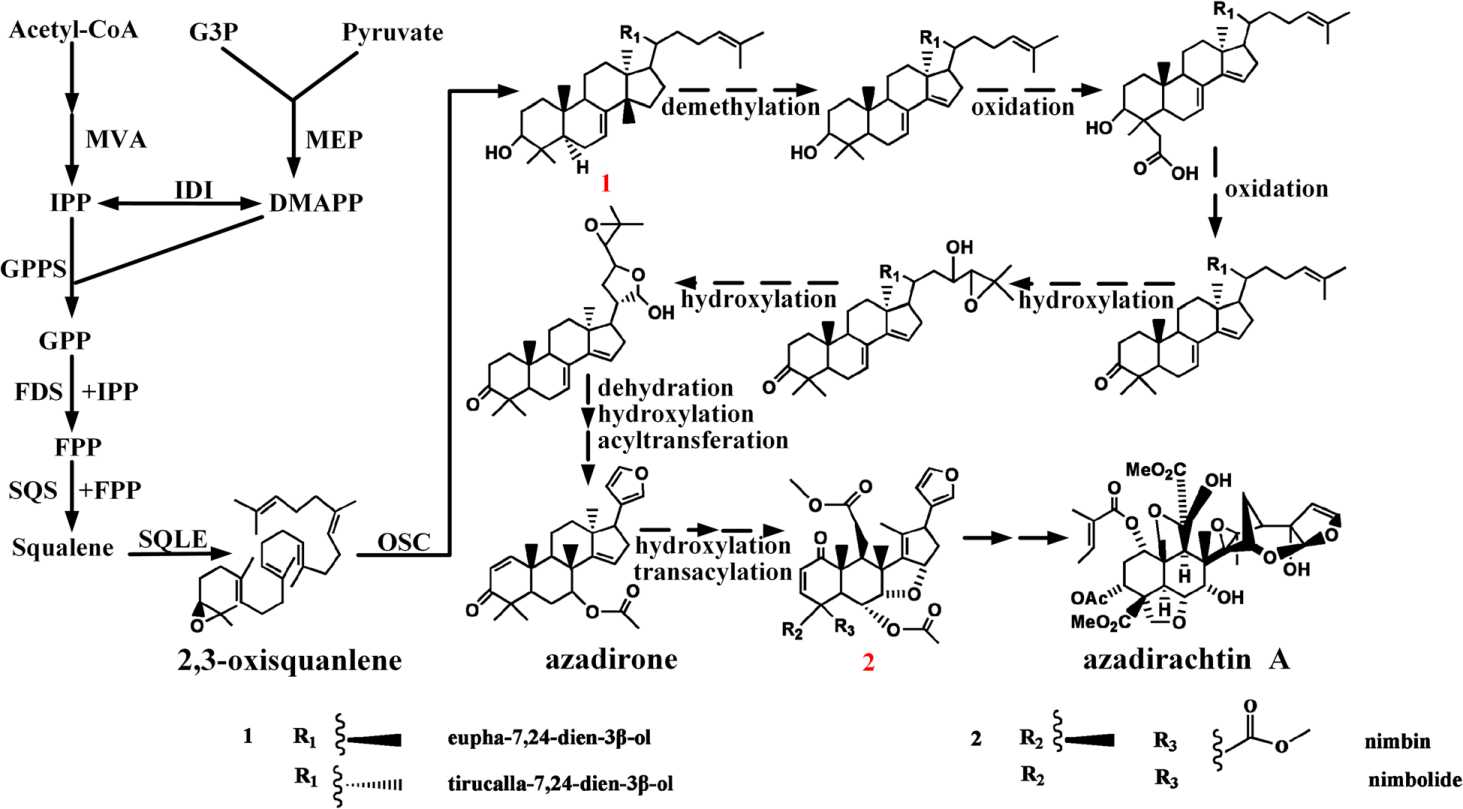
Hypothetical pathway of azadirachtin biosynthesis in *A. indica*. Isopentenyl-diphosphate δ-isomerase (IDI); Geranyl diphosphate synthase (GPPS); Farnesyl diphosphate synthase (FDS); Squalene epoxidase (SQLE); 2,3-oxidosqualene cyclase (OSC); G3P: 3-phosphoglyceraldehyde; MVA: mevalonate; MEP: methylerythritol phosphate; IPP: isopentenyl pyrophosphate; DMAPP: γ-dimethylallyl pyrophosphate; GPP: geranyl pyrophosphate; FPP: farnesyl pyrophosphate. The pathway of 2,3-oxidosqualene biosynthesis is connected by solid lines and arrows. The putative biosynthetic pathway of azadirachtin A is connected by dashed lines and arrows. Isomers are numbered in red and detailed structures are shown in the figure

While the three steps involved in azadirachtin A biosynthesis have been characterized, the rest of the downstream pathway is still unexplored. Based on the metabolites in the Neem Metabolite Structure Database [26] as well as the distribution of metabolites (Table S1) in neem tissues, a putative biosynthetic pathway for azadirachtin A (Fig. 1) was proposed. Five kinds of putative enzymes, oxidosqualene cyclase (OSC), cytochrome P450 (CYP450) [27], alcohol dehydrogenase (ADH) [28], acyltransferase (ACT), and esterase (EST) [29] were proposed in the putative biosynthetic pathway. Candidate mining was performed through the workflow of gene mining (Figure S1). Extensive bioinformatic analysis of unigenes involving phylogenetic analysis, domain prediction, and molecular docking further provided 22 candidates (Table 1) for the putative biosynthetic pathway, including 1 OSC unigene, 2 ADH unigenes, 12 CYP450 unigenes, 2 ACT unigenes, and 5 EST unigenes. Among them, 3 transcripts encoding the complete AiOSC1 and homologs of MaCYP71CD2 and MaCYP71BQ5 were also found in our study. Unigene containing the complete open reading frame (ORF) encoding the homolog of MaCYP71BQ5 [18] was first reported. Quantitative real-time PCR and full-length PCR cloning were used for verifying unigene expression level (Table S2) (Fragments per kilobase of transcript per million mapped reads (FPKM)) and transcript sequence accuracy. The obtained candidates could be used as an important resource to investigate the catalysts responsible for essential biochemical reactions in azadirachtin A biosynthesis as well as triterpenoid metabolism in closely related species of neem.

**Table 1:** Summary of candidate genes involved in azadirachtin A biosynthesis in neem

Classification	Unigene	Gene accession number	Homologs
OSC	transcript/14449	gi|443299067	AiOSC1*
ADH	transcript/18833	gi|572153023	cinnamyl-alcohol dehydrogenase
	transcript/19291	gi|572153023	cinnamyl-alcohol dehydrogenase
CYP450	transcript/17636	gi|225458053	CYP83B1
	transcript/17854	gi|641826901	CYP77A3
	transcript/16057	gi|567902124	flavonoid 3′-monooxygenase
	transcript/17284	gi|567889747	CYP89A2
	transcript/16777	gi|590722535	brassinosteroid-6-oxidase
	transcript/17001	gi|590722535	brassinosteroid-6-oxidase
	transcript/16577	gi|568834016	flavonoid 3′-monooxygenase
	transcript/16950	gi|645239614	cytokinin trans-hydroxylase
	transcript/17057	gi|567868115	flavonoid 3′-monooxygenase
	transcript/16971	gi|568825869	MaCYP71CD2*
	transcript/16742	gi|568830413	MaCYP71BQ5*
	transcript/16198	gi|568868580	CYP51G1
ACT	transcript/17792	gi|567873443	BAHD acyltransferase
	transcript/18214	gi|641830965	BAHD acyltransferase
EST	transcript/19188	gi|568822600	SGNH_plant_lipase
	transcript/19882	gi|568867507	Acetyl esterase/lipase
	transcript/19697	gi|225440163	Acetyl esterase/lipase
	transcript/19748	gi|641846434	Acetyl esterase/lipase
	transcript/18100	gi|568835134	pectin acetylesterase

Homologs with * represent the identified gene in the previous report [18]

## Results

**Fig. 2:**
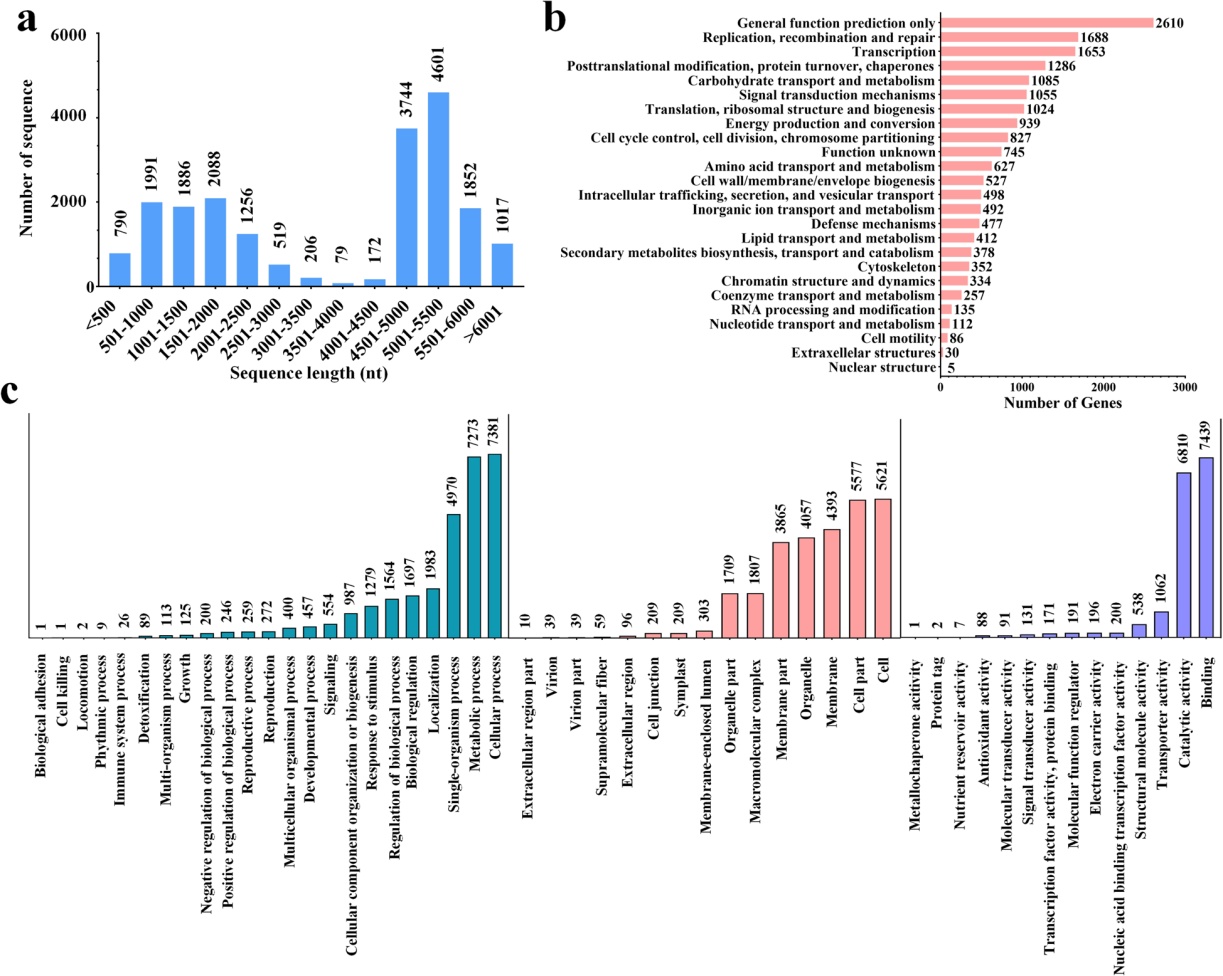
Analysis of size distribution and gene classification. a) the size distribution of de novo assembled unigenes of *A. indica*, b) COG functional classification, and c) distribution of GO terms assigned to unigenes of assembled cDNA library

To generate a comprehensive overview of neem transcriptome, total RNAs were extracted from leaves, fruits (containing seeds), roots, stems, and flowers. To obtain the transcriptome data, we used the hybrid-seq technology that combines Illumina HiSeq and PacBio SMRT sequencing data and corrects the errors in long reads with short reads [24]. Different neem tissues (leaves, flowers, stems, fruits (containing seeds) and roots) were sequenced separately using Illumina HiSeq platform and generated 41.14, 41.35, 40.60, 40.59 and 40.64 million clean reads, respectively. PacBio SMRT platform produced 6.75 million clean reads. After calibration with short reads from Illumina platform, the assembled unigenes were corrected with an N50 of 5076 bp and mean length of 3607 bp (Table 2). The obtained assembly was 2.5 times longer than in the previous report [14]. The length of these unigenes ranged from 500 to 6001 bp. The majority (over 55.5%) of reads were distributed in the range of 4501 bp and above (Fig. 2a).

**Table 2:** Summary of Illumina and PacBio output data quality and assembled sequences of libraries of neem

Sequencing Platform	Sample	Reads Number (M)	Clean Bases (G)	Q20 (%)	Q30 (%)	Total number of unigene	Total length of unigene (bp)	Mean length of unigene (bp)	N50	GC (%)
Ilumina HiSeq	**Leaf**	41.14	6.17	96.95	92.30	50394	82508501	1380	2201	40.64
	**Flower**	41.35	6.20	97.29	93.06	62426	95564612	1530	2335	40.13
	**Stem**	40.60	6.09	97.29	93.11	65762	98612970	1499	2372	40.39
	**Fruit**	40.59	6.09	97.45	93.39	66668	81799852	1226	2077	41.93
	**Root**	40.64	6.10	97.44	93.43	45459	57083335	1255	2100	41.10
Hiseq summary	–	–	–	–	–	113008	175268545	1550	2599	40.99
PacBio SMRT	**Mixed tissue**	6.75	11.28	–	–	22884	82035635	3584	5068	43.55
Calibrated PacBio by Illumina		–	–	–	–	20201	72872459	3607	5076	43.55

Q20 and Q30 on Illumina platform correspond to the predicted base call error rate of 1% and 0.1%, respectively

N50: The minimum contig length needed to cover 50% of the transcriptome

To confirm the accuracy of the hybrid-seq (FPKM) results, we selected 10 unigenes and used qRT-PCR to determine their relative expression (Figure S2). The qRT-PCR and FPKM results were consistent except for transcript/19882 and transcript/16577. Their expression from RNA-seq (FPKM) and relative expression level by qRT-PCR in root and stem were inconsistent. The inconsistency between qRT-PCR and FPKM in transcriptome analysis has also been reported by other researchers. In the study by Zhang [30], the FPKM expression levels of 8 out of 58 genes from transcriptome were inconsistent with their qRT-PCR results. In another study comparing the gene expression fold changes in the samples of *Metarhizium acridum* CQMa102, approximately 86.2% of the genes showed consistent results between RNA-sequencing and qRT-PCR data [31]. The inconsistency between FPKM and qRT-PCR may result from multiple reasons. Although qRT-PCR and RNA-seq are both used to measure gene expression, the unit of measurement [32] as well as the computing method are different for FPKM and qRT-PCR. Many factors affect the accuracy of FPKM and qRT-PCR. Bias in PCR amplification [33] and RNA-seq library preparation [34] and sequencing adds noise to the RNA-seq data. The quality of the mRNA, amplification efficiency, and the choice of reliable internal controls referred to as reference genes affect the accuracy of qRT-PCR [35]. Therefore, the consistency of transcript/16577 and transcript/19882 between qRT-PCR and FPKM was acceptable. Further examinations need to be performed on these two unigenes.

Ten unigenes were cloned by PCR using primers listed in Additional file 1. According to the sequencing results of the cloned unigenes, each of them was 100% identical to the sequences obtained from hybrid-seq platform. Among them, 4 unigenes attracted our attention since they contained a complete ORF of the genes. One of them (transcript/14554) was annotated as the neem NADPH-cytochrome P450 reductase 2. The cloned transcript/14449 was found to encode an OSC consisting of 760 amino acids. The length of two CYP450 unigenes (transcript/16971 and transcript/16742) was 1536 bp and 1527 bp, and they encoded proteins consisting of 511 and 508 amino acids, respectively. Detailed sequencing results indicated that the unigenes in our study had good accuracy and were therefore reliable for further analyses.

### Functional annotation and classification of unigenes

A total of 19,907 unigenes (98.54% of 20,201 unigenes) were annotated in at least one database (Table S3). The annotated unigenes were compared to known nucleotide sequences of other plant species. They best matched to the known nucleotide sequences from *C. sinensis* (52.43%), *Citrus clementine* (23.49%), *Theobroma cacao* (2.44%), *Vitis vinifera* (2.4%), and others (19.23%).

Clusters of Orthologous Groups of proteins (COG) and Gene Ontology (GO) classification were used to further evaluate the completeness and effectiveness of the neem annotation. All 17,634 unigenes (87.3% of 20,201 unigenes) were classified into 25 functional COGs (Fig. 2b). 2610 unigenes (14.8% of the total 17,634 classified unigenes) were categorized into general function prediction only cluster, which formed the largest group, whereas the clusters for replication, transcription, recombination, and repair followed closely. Although only 378 unigenes were categorized into the “Secondary metabolites biosynthesis transport and catabolism” cluster, they may play important roles in providing precursors for secondary metabolite biosynthesis.

A total of 13,453 assembled unigenes (66.6% of 20,201 unigenes) were assigned at least one of the 55 GO terms (Fig. 2c). These unigenes were predominantly assigned to metabolic process (GO:0008152) and cellular process (GO:0009987). The unigenes categorized in the molecular function category were predominantly associated with catalytic activity (GO:0003824) and binding functions (GO:0005488). The unigenes categorized in the cellular component category were predominantly associated with membrane (GO: 0016020), cell part (GO: 0044464), and cell (GO: 0005623). These findings showed that the main COG and GO classifications for the fundamental biological processes were identified.

The Kyoto Encyclopedia of Genes and Genomes (KEGG) pathway database was used to systematically evaluate the gene biological functions in different pathways. A total of 16,778 unigenes were matched to the database and assigned to 135 KEGG pathways (Table S4). Metabolic pathways (3590 unigenes, 21.4%) and biosynthesis of secondary metabolites (1560 unigenes, 9.3%) were the two dominant categories. In the biosynthesis of secondary metabolites category (Table S5) in neem, subcategories of flavonoid biosynthesis, terpenoid backbone biosynthesis (Table S6), steroid biosynthesis, sesquiterpenoid and triterpenoid biosynthesis (Table S7), diterpenoid metabolism, and carotenoid biosynthesis were included. There were 242 unigenes involved in the metabolic pathways of terpenoids and polyketides.

### Differential expression analysis of unigenes in neem

In order to find candidate genes in the azadirachtin A biosynthesis pathway, all transcripts had been identified, annotated, and mapped in different pathways. Genes with a higher expression in the tissues with high azadirachtin A content, such as fruits and leaves, are more likely to be the involved in azadirachtin A biosynthesis. The expression level of unigenes was calculated by the FPKM method. The upregulated DEGs in leaf and fruit were unigenes which higher-expressed in leaf or fruit compared to the other tissues. Up-regulated DEGs in fruit and leaf were 219 and 397, respectively. These DEGs were used for mining candidates involved in azadirachtin A biosynthesis.

### First screening of candidate genes involved in azadirachtin a biosynthesis

According to the putative azadirachtin A pathway in Fig. 1, tirucalla-7,24-dien-3β-ol is assumed as the scaffold formed from 2,3-oxidosqualene. A few steps such as hydroxylation and furan ring formation occur after scaffold formation. The hydroxyl groups are then either oxidized to acid or acylated or esterified to esters, forming limonoid compounds like azadirone or nimbin. Azadirachtin A is finally obtained after modifications on azadirone or nimbin. ADH, CYP450, ACT, and EST are supposed to be involved in azadirachtin downstream pathway and thus their encoding-unigenes were chosen as candidates.

**Fig. 3:**
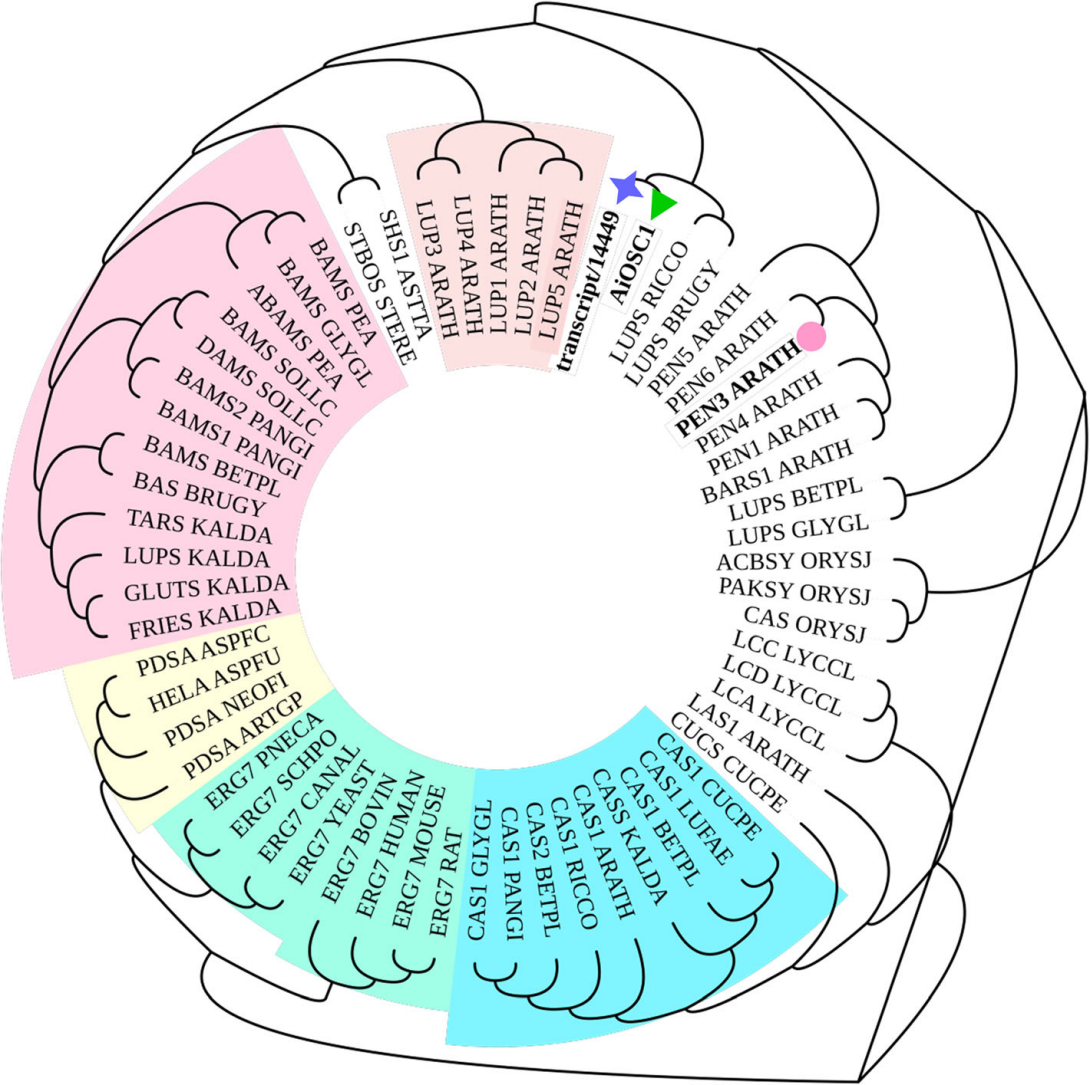
Phylogenetic tree of candidate OSC from neem transcriptome. Functionally characterized OSCs from other plant species including the previously characterized tirucalla-7,24-dien-3β-ol synthases from *A. thaliana* (PEN3) (bold, marked with pink circle) and AiOSC1 (bold, marked with green triangle) identified previously. Candidate OSC chosen for further analysis is displayed in bold and marked with a purple star. The phylogenetic tree was constructed by MEGA V7 and formatted using iTOL

As a triterpenoid, the first step of azadirachtin A biopathway was the formation of its scaffold catalyzed by OSC. Among all unigenes involved in terpenoid biosynthesis, 8 detected unigenes were annotated as OSC, including transcript/1784, transcript/1866, transcript/8176, transcript/8892, transcript/9751, transcript/14584, transcript/19700, and transcript/14449. Among them, only transcript/14449 expressed higher in fruit. After phylogenetic analysis (Fig. 3) of transcript/14449 with several characterized OSCs from other plants, transcript/14449 was grouped with AiOSC1 [18], a newly characterized OSC from neem, catalyzing the formation of tirucalla-7,24-dien-3β-ol. The other unigenes were grouped with cycloartenol synthase. After DNA sequence analysis with AiOSC1(Figure S3), transcript/14449 is 100% identical to AiOSC1, which means that these two genes were the same gene. It indicated that transcript/14449 could be a candidate gene for producing the azadirachtin A scaffold.

Among all the DEGs in fruit and leaf transcriptome data, DEGs encoding ADH, CYP450, ACT and EST were discovered. There were sixteen DEGs encoding ADH. Four up-regulated DEGs encoding ADH were selected for further screening. As for CYP450, sixteen DEGs encoded CYP450 and ten DEGs were selected. Similarly, thirteen and nineteen DEGs encoded ACT and EST respectively and there were four and twelve DEGs up-regulating in leaf and fruit tissues, respectively. DEGs with same sequence or sequences within 150 amino acids were excluded in further screening.

Among all the DEGs in fruit and leaf transcriptome data, DEGs encoding ADH, CYP450, ACT, and EST were identified. There were 16 DEGs encoding ADH, 4 upregulated DEGs encoding ADH were selected for further screening. There were 16 DEGs encoding CYP450, 10 out of these DEGs were selected. Similarly, 13 and 19 DEGs encoded ACT and EST, respectively. There were 4 and 12 DEGs upregulated in leaf and fruit tissues, respectively. DEGs with the same sequence or sequences coding for proteins of length less than 150 amino acids were excluded from further screening.

### Further screening of the other four enzymes through phylogenetic analysis and domain prediction

**Fig. 4:**
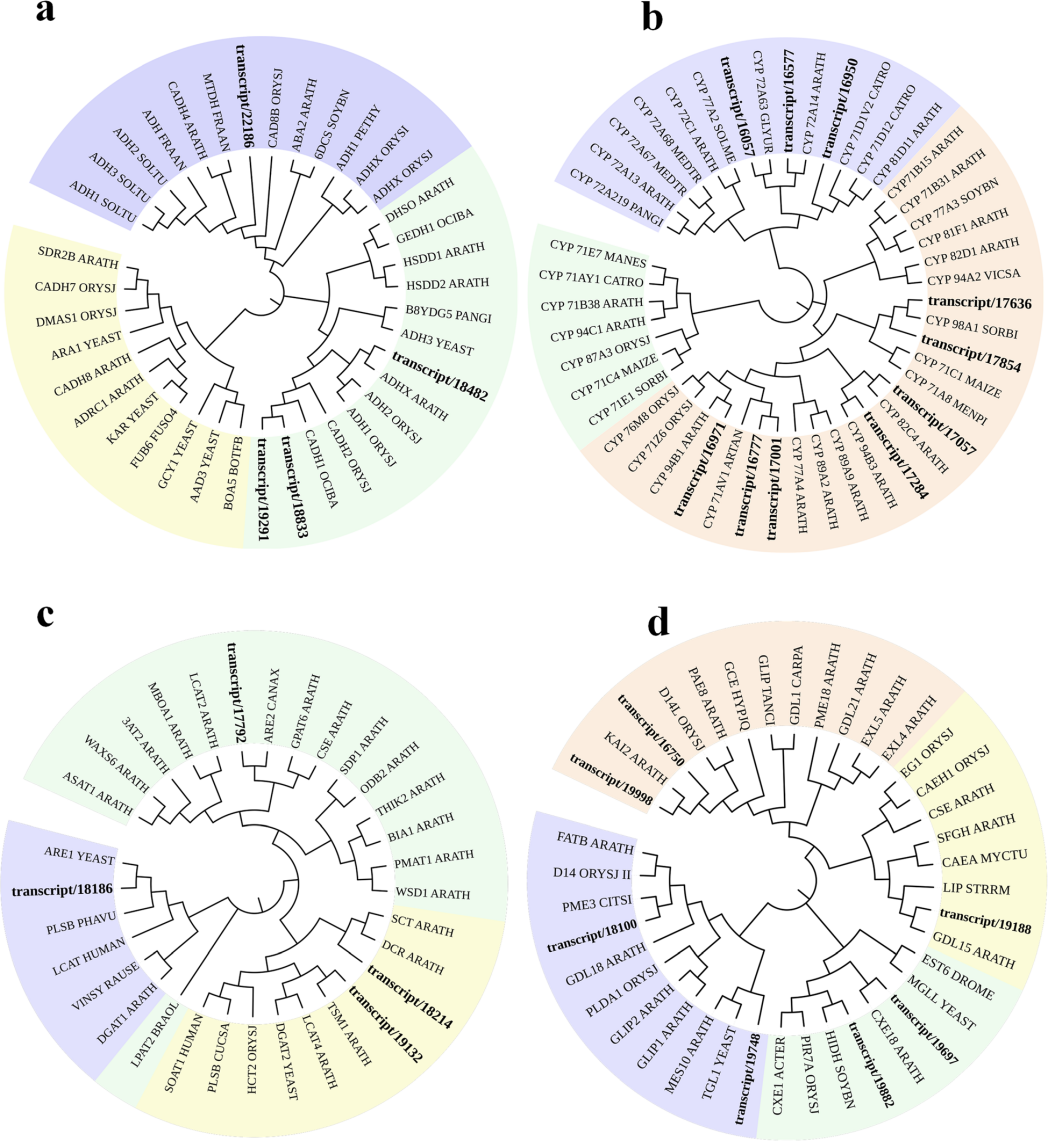
Phylogenetic analysis of a) alcohol dehydrogenase, b) CYP450, c) acyltransferase, and d) esterase candidates. Neighbor-joining trees were constructed for four types of *A. indica* candidate enzymes along with corresponding proteins identified from other plant species. Candidates are marked in bold

Phylogenetic analysis (Fig. 4) and protein domain prediction were used for further screening of these DEGs. Both, transcript/22186 and transcript/18833, were grouped with cinnamyl-alcohol dehydrogenase 4 (CADH4) [36] and transcript/19291 was grouped with CADH1. CADHs catalyze the biosynthesis of cinnamaldehyde from cinnamyl alcohol. Transcript/18482 was grouped with ADHX [37] which showed activity to primary and secondary alcohols. Transcript/18833 and transcript/19291 contained the PLN02514 domain that was also found in CADH [38]. Transcript/22186 contained the nsLTP2 [39] domain that is present in non-specific lipid-transfer protein. Transcript/18482 contained the GxGxxG motif [40] found in S-(hydroxymethyl) glutathione dehydrogenase. Transcript/18833 and transcript/19291 were selected as candidates for further examination.

According to the phylogenetic analysis of unigenes encoding CYP450, 5 transcripts (transcript/16057, transcript/16577, transcript/16950, transcript/16777, and transcript/17001) were grouped with members in CYP71 [41] and CYP72 [42] clades, whose members are reported to be involved in terpenoid biosynthesis. Transcript/16971 was grouped with CYP94B1 [43], an enzyme catalyzing the hydroxylation at C12 of jasmonyl-L-amino acid. Transcript/17284, transcript/17057, transcript/17636, and transcript/17854 were grouped in another clade. Transcript/17057 fell into a group with CYP82C4 [44], an enzyme hydrolyzing xanthotoxin (8-methoxypsoralen) into 5-hydroxyxanthotoxin. Transcript/17284 was grouped with CYP94B3 [45], this revealed that transcript/17284 may act as a hydroxylase. Transcript/17636 and transcript/17854 were classified into a group with uncharacterized CYP98A1.

According to CYP450 domain analysis, transcript/17636 and transcript/16971 contained the same CypX domain [46] as CYP81B1. The P450-cyclo_AA_1 domain in transcript/16950 was also found within cytokinin trans-hydroxylase [47]. The PLN00168 domain [48] was found in transcript/17824 and transcript/19854. The PLN02687 domain (a domain in flavonoid 3′-monooxygenase [49]) was also contained by transcript/16057, transcript/16577, and transcript/17057. Transcript/16777 and transcript/17001 contained the PLN02774 domain, which is often found in brassinosteroid-6-oxidase [50]. Therefore, 5 DEGs (transcript/16057, transcript/ 16,577, transcript/17057, transcript/16777, and transcript/17001) contained domains found within CYP450 oxidases and the others contained domain found within CYP450 hydroxylase.

Among all ACT DEGs, transcript/18186 was grouped with ARE1, the enzyme encoding sterol *O*-acyltransferase [51]. Transcript/18214 fell into a subclade with a DCR, a member of BAHD acyltransferase from *A. thaliana* involved in cutin biosynthesis [52]. Transcript/17792 was grouped with ARE2, a sterol O-acyltransferase from *Candida albicans* [53]. Transcript/19132 and TSM1 [54] were in a group which reveal that transcript/19132 was likely to be methyltransferase. Through domain analysis, transcript/17792 and transcript/18214 were shown to contain the HXXXD domain that is often found in the BAHD ACT family [52]. Transcript/19132 contained the domain PLN02177, also found in glycerol-3-phosphate acyltransferase [55]. Transcript/18186 and eukaryotic initiation factor 4B [56] had the same eIF-4B domain. Therefore, after combining phylogenetic analysis and domain prediction, transcript/17792 and transcript/18214 were selected as candidates of ACT involved in azadirachtin A biosynthesis.

Upon phylogenetic analysis of all EST DEGs, transcript/19998 and transcript/16750 were grouped with KAI2 [57]. KAI2 has been reported to be involved in seed germination and did not show esterase activity. Transcript/19188 was divided into a subclade with *A. thaliana* GDL15, that belonged to GDSL-like [58] lipase/ acylhydrolase superfamily and displayed hydrolytic activity with esters. Transcript/18100 was grouped with PME3, a pectinesterase catalyzing the hydrolysis of (1,4)-α-D-galacturonosyl methyl ester [59]. Transcript/19748 formed a tight subclade with TGL1, which is a sterol esterase mediating the hydrolysis of steryl esters [60]. Transcript/19882 and transcript/19697 were in the same group as HIDH [61] and CXE18, respectively and these two enzymes show activity to carboxylic esters. The conserved domain analysis of EST candidates presented that transcript/19188 contained the Ser-His-Asp (Glu) triad found in an SNGH plant lipase [62]. Transcript/19882, transcript/19697, and transcript/19748 had the AES domain [63] which is also contained within acetyl esterase/lipase. PAE domain [64] in transcript/18100 was also found in pectin acetylesterase while transcript/16750 and transcript/19998 contained the plant pectinesterase inhibitor domain PLN02201. Therefore, transcript/16750 and transcript/19998 were removed from the list of candidates after phylogenetic and domain analysis.

### Molecular docking analysis of CYP450s

**Fig. 5:**
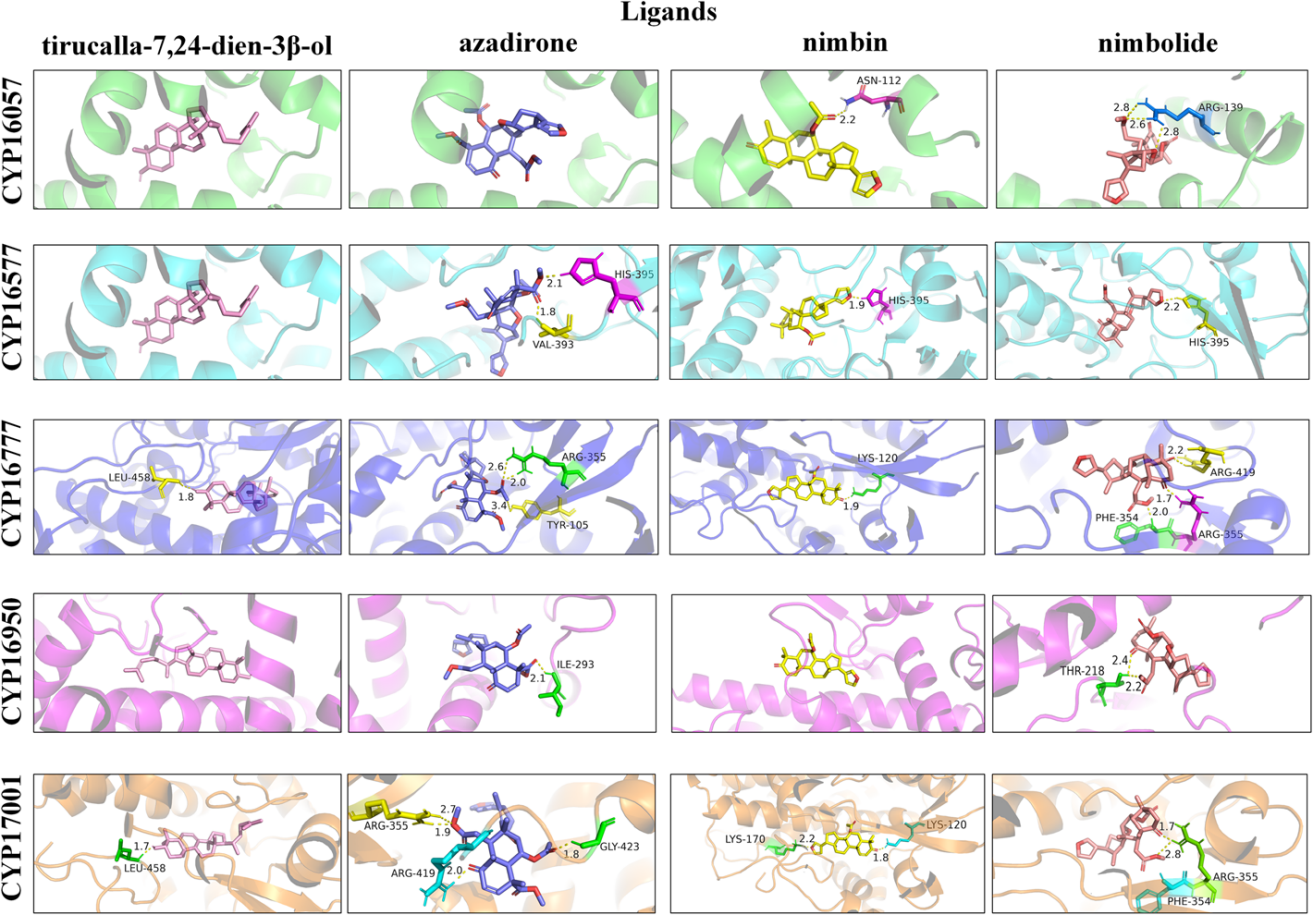
Molecular docking analysis of CYP450s putatively involved in azadirachtin A biosynthesis. Interaction of 5 CYP450s’ docked regions with four ligands (tirucalla-7,24-dien-3β-ol, azadirone, nimbin, and nimbolide) are shown in the figure. CYP450s and the four ligands are represented in different colors. The zoomed blocks show the interacting orientation of functional moieties of tirucalla-7,24-dien-3β-ol, azadirone, nimbin, and nimbolide within the cavity of protein. The hydrogen bonds formed between residue and ligand are displayed in yellow and the distances (Å) are also shown

The active site prediction in the 5 CYP450s was performed and the results are listed in Table S8. To further analyze the interactions between CYP450s and the four ligands, molecular docking was performed with Autodock 4.0. The details of docking are listed in Table S9 and specific interactions are displayed in Fig. 5. Analysis revealed that binding energy was lowest in case of CYP16057 docked with tirucalla-7,24-dien-3β-ol forming zero hydrogen bond. However, azadirone and nimbolide formed stable complexes with CYP16057 with one hydrogen bond with − 7.20 and − 6.40 kcal/mol of binding energies (Fig. 5 and Table S9), respectively. The docking analysis for CYP16577 revealed that among all the ligands, binding energy was lowest for tirucalla-7,24-dien-3β-ol and nimbin with − 10.07 and − 9.83 kcal/mol, respectively, forming zero and two hydrogen bonds, respectively. Docking of CYP16577 with triterpenoids showed interaction through one hydrogen bond with binding energy of − 9.42 and − 9.16 kcal/mol for azadirone and nimbolide, respectively (Fig. 5 and Table S9). The binding energy of CYP16777 docked with azadirone and tirucalla-7,24-dien-3β-ol was − 9.96 and − 9.75 kcal/mol, respectively, forming one hydrogen bond each. However, nimbolide and nimbin formed stable complexes with three and two hydrogen bonds respectively, indicating that the conformation of nimbolide is best suitable for CYP16777 when the number of hydrogen bonds formed between protein and ligand is set as the criterion. The hydrogen bonds between nimbolide and CYP16777 are formed at PHE354, ARG355, and ARG419, with ligand moiety at different positions (Table S9) with varying bond length (Fig. 5). The docking analysis for CYP16950 revealed that among all the ligands, binding energy was lowest for tirucalla-7,24-dien-3β-ol and azadirone with − 8.31 and − 7.90 kcal/mol without forming hydrogen bond. However, nimbin and nimbolide formed stable complexes with CYP16950 with one hydrogen bond with − 6.32 and − 6.66 kcal/mol binding energies, respectively (Fig. 5 and Table S9). Docking of CYP17001 with triterpenoids showed interaction through only one hydrogen bond with the lowest binding energy of − 10.11 kcal/mol for tirucalla-7,24-dien-3β-ol. Both, azadirone and nimbolide, formed stable complexes with CYP17001 through two hydrogen bonds. The hydrogen bonds between nimbin and CYP17001 are formed at ARG355, ARG419, and GLY423 with the binding energy of − 9.82 kcal/mol (Fig. 5 and Table S9).

## Discussion

Tirucalla-7,24-dien-3β-ol was confirmed to be the scaffold of azadirachtin A. Nimbin, nimbolide, and azadirone are three important compounds isolated from neem. They were proposed as intermediates in azadirachtin A pathway. According to the docking analysis of five CYP450s with all the ligands, CYP16057, CYP16577, CYP16950, and CYP17001 showed strongest binding with tirucalla-7,24-dien-3β-ol. CYP16777 could more easily bind with azadirone. Three residues in CYP16777 and CYP17001 formed stable hydrogen bonds with nimbolide and nimbin, respectively. All the docking results indicate the priority of reactions between five CYP450s and four ligands. It also provided a theoretical basis for further functional assays of these CYP450s. The residues in proteins forming hydrogen bond with ligands also led to identification of sites for mutation analysis to improve the catalytic ability of these CYP450s.

**Fig. 6:**
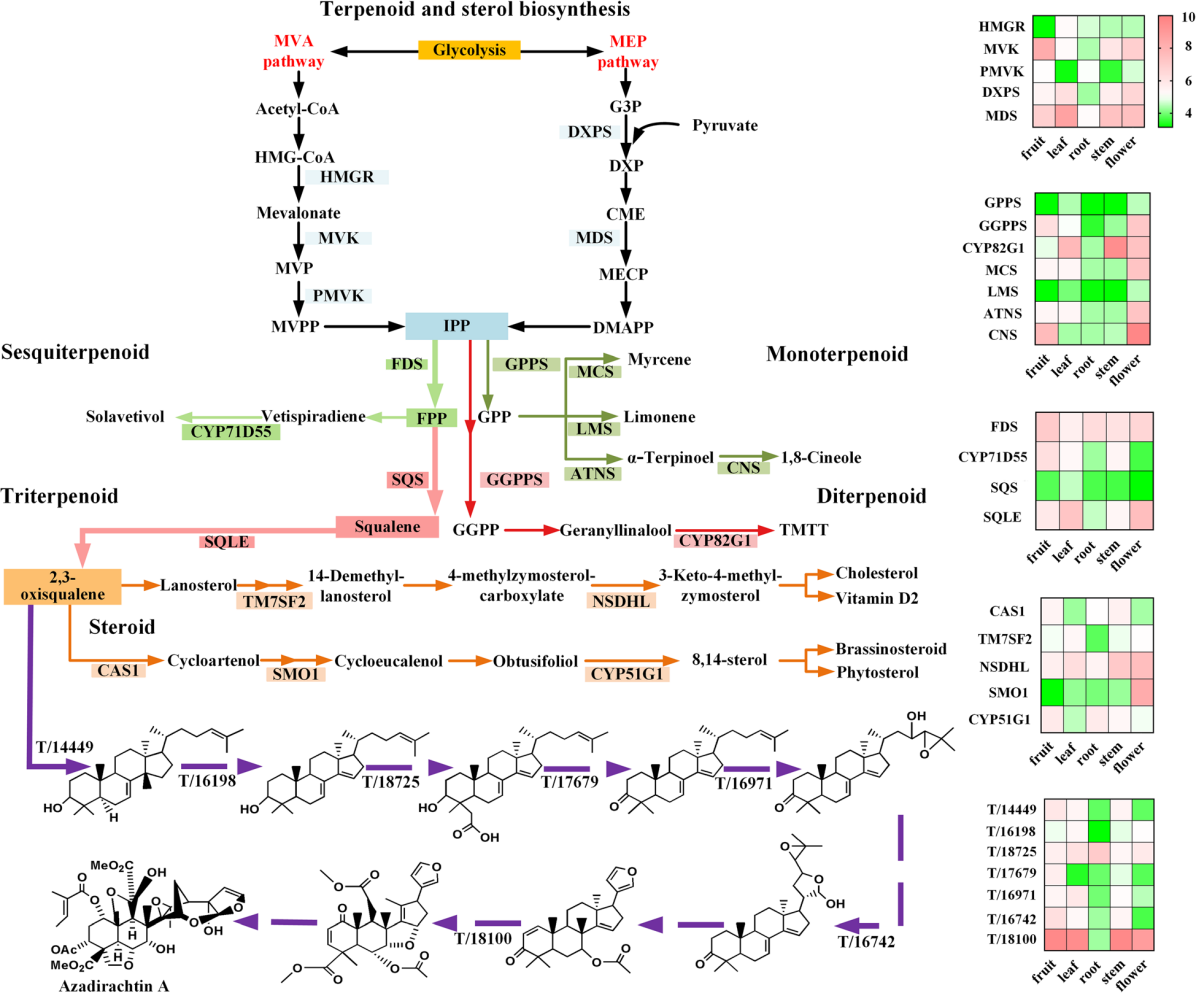
Mapping of unigenes related to secondary metabolites in *A. indica*. Tissue abundance and relative expression patterns are color coded and represented by bars, respectively. G3P: 3-phosphate glyceraldehyde; DXP: 1-Deoxy-D-xylulose 5-phosphate; MVP: 5-phosphomevalonate; MVPP: (R)-5-diphosphomevalonate; IPP: isopentenyl pyrophosphate; FPP: farnesyl pyrophosphate; GPP: geranyl pyrophosphate; GGPP: geranylgeranyl pyrophosphate; HMG-CoA: 3-hydroxy-3-methyl-glutaryl-CoA; PCME: 2-phospho-4-2-C-methyl-D-erythritol; MECP: 2-C-methyl-D-erythritol 2,4-cyclodiphosphate; HMBDP: 1-hydroxy-2-methyl-2-butenyl 4-diphosphate; TMTT: (E,E)-4,8,12-trimethyltrideca-1,3,7,11-tetraene; DXPS: 1-deoxy-D-xylulose-5-phosphate synthase; MDS: 2-C-methyl-D-erythritol 2,4-cyclodiphosphate synthase; HMGR: Hydroxymethylglutaryl-CoA reductase; MVK: Mevalonate kinase; PMVK: Phosphomevalonate kinase; GPPS: Geranyl diphosphate synthase; MCS: Myrcene/ocimene synthase; LMS: (R)-limonene synthase; ATNS: (−)-alpha-terpineol synthase; CNS: 1,8-cineole synthase; TM7SF2: Delta (14)-sterol reductase; NSDHL: Sterol-4-alpha-carboxylate 3-dehydrogenase; SMO1: Methylsterol monooxygenase 1; FDS: Farnesyl diphosphate synthase; GGPPS: Geranylgeranyl diphosphate synthase; SQS: Squalene synthase; SQLE: Squalene monooxygenase; CAS1: Cycloartenol synthase

The expression levels of unigenes involved in secondary metabolite pathways including three terpenoid and two sterols, and putative azadirachtin A downstream pathway were analyzed based on the KEGG annotation and the FPKM method (Fig. 6 and Table S10). The molecular docking results of candidate proteins involved in azadirachtin A biosynthetic pathway in Fig. 6 were provided in Table S11. Among all the unigenes, 13 were found to be related to the MVA pathway and 38 unigenes were found to be related to the MEP pathway (Table S6). Some of them (unigenes encoding mevalonate kinase (MVK), 1-deoxy-D-xylulose-5-phosphate synthase (DXPS), and 2-C-methyl-D-erythritol 2,4-cyclodiphosphate synthase (MDS)) were expressed at higher levels in leaf and fruit. Geranyl pyrophosphate (GPP), catalyzed by geranyl diphosphate synthase (GPPS) was the common intermediate of monoterpenoids. Four enzymes involved in myrcene, limonene, and terpineol biosynthetic pathways were highly expressed in flower and fruit. Unigenes encoding geranylgeranyl diphosphate synthase (GGPPS) and CYP82G1, involved in (E, E)-4, 8, 12-trimethyltrideca-1, 3, 7, 11-tetraene (TMTT) biosynthesis, were expressed at higher levels in flower and stem.

Farnesyl diphosphate synthase (FDS) catalyzes the formation of farnesyl pyrophosphate (FPP) from IPP, the unigene encoding FDS is expressed at the highest level in fruit followed by root and flower. Solavetivol pathway is one of the sesquiterpenoid pathways, and the unigene encoding solavetivol synthase (CYP71D55) is expressed at higher levels in fruit and leaf. In triterpenoid biosynthesis, two FPPs under the continuous catalysis of squalene synthase (SQS) and squalene epoxidase (SQLE) form 2,3-oxidosqualene. SQS and SQLE encode unigenes that are expressed the highest in leaf. The expression level of the unigene encoding cycloartenol synthase (CAS1) in different tissues was in the order of fruit > stem > root. Methylsterol monooxygenase (SMO1) and sterol-4-alpha-carboxylate 3-dehydrogenase (NSDHL) were expressed at the highest level in flower. Unigenes encoding delta (14)-sterol reductase (TM7SF2) and CYP51G1 were highly expressed in leaf and fruit, respectively.

The expression level of unigenes involved in putative azadirachtin A downstream pathway has been presented. Transcript/14449 encoding the first enzyme in azadirachtin A downstream pathway is expressed at the highest level in fruit followed by in leaf. After scaffold synthesis, the methyl group at C14 is removed. This is catalyzed by the enzyme encoded by transcript/16198, that is highly expressed in leaf. Alcohol at C3 is continuously oxidized into C3 ketone group by transcript/18725 and transcript/17679 and forms common compounds [65] isolated from Meliaceae family. These two unigenes expressed highest in root and fruit, respectively. Next important step involved in azadirachtin A is the formation of the furan ring, the two CYP450s catalyzing its formation were isolated from *M. azedarach* and *C. sinensis* [18]. Two transcripts (transcript/16971 and transcript/16742) in our study were found to be homologs of the two identified CYP450s and might produce melianone [66] from its precursor. Transcript/16971 and transcript/16742 expressed highest in leaf and fruit, respectively. With some unknown enzymes, melianone is further modified into a compound with furan ring and C7-OH. The insecticidal C7-hydroxylated compound [67] is esterized by transcript/18100 (highly expressed in fruit and leaf) and further forms compounds like nimbin or nimbolide after some modifications. However, reactions between nimbin or nimbolide and azadirachtin A are still unclear.

Upon bioinformatic analysis of these DEGs, 2 transcripts (transcript/18833 and transcript/19291) encoding ADH, 12 transcripts encoding CYP450, 2 ACT transcripts (transcript/17792 and transcript/18214), and 5 transcripts encoding EST, were selected as candidates in the azadirachtin A downstream pathway. Some DEGs were removed from the library after phylogenetic analysis and domain prediction and some non-encoding DEGs were also deleted. DEGs were also selected even though they were not expressed at higher levels in fruit and leaf, for example, transcript/16198, transcript/16742, and transcript/16950. Transcript/16198 was annotated to encode sterol 14-demethylase, that removes the methyl group from C14 of sterol. Transcript/16742 encodes a protein with 509 amino acids and it was 98% identical to MaCYP71BQ5 (Figure S4). MaCYP71BQ5 is the CYP450 found to be involved in melianol formation. Researchers could only get a fragment of its homolog (AiCYP71BQ5) from neem [18], whereas, our transcript/16742 contained the complete ORF of AiCYP71BQ5.

The increase in terpenoid precursor leads to the higher production of terpenoid. In the case of artemisinic acid production, improvement of terpenoid precursor by engineering the MVA pathway resulted in an increase in yield by 500 times [68]. Thus, the upregulated DEGs in the MVA or MEP pathway and the 2,3-oxidosqualene biosynthetic pathway could be used as building blocks in the construction of azadirachtin A precursor biosynthetic pathway in the future.

Although reaction types and key enzymes were partially proposed based on the structural differences between intermediates in our putative azadirachtin A pathway, some information was still missing. For instance, we could not find the enzyme that catalyzes the hydroxylation reaction at C7 site. Further, the order of reactions downstream of azadirachtin A was not clear. Neither the number of reactions nor the catalysis type were characterized. These limitations of pathway lead to insufficient mining of the neem transcriptome data. This might be one of the reasons for slow progress in azadirachtin pathway exploration even though numerous neem genome and transcriptome data are available.

## Conclusions

In conclusion, the multi-tissue transcriptome analysis revealed five types of genes potentially involved in azadirachtin A downstream pathway and their respective transcript levels. It also indicated that the neem tree genome encodes a high number of terpene or limonoid biosynthetic genes. Finally, 22 unigenes encoding enzymes including OSC, ADH, CYP450, ACT, and EST were selected as candidates involved in azadirachtin A downstream pathway. This is the first report on hybrid-seq transcriptome profiling analysis of *A. indica*. The obtained unigenes may provide a valid and diverse candidate pool for the study of selective modification by the functional groups in the triterpenoid or limonoid skeleton as well as for the study of convergent evolution in secondary metabolism.

## Methods

### Plant materials

Fresh and healthy tissues (from root, leaf, stem, flower, and fruit containing seed) were randomly sampled from a neem (*Azadirachta indica, A. indica*) tree grown in the Liufang Yuan park (110°33ʹE 20°05ʹN) in Hainan University, Meilan District, Haikou, Hainan Province of China, followed by their transcriptome analysis. All samples for RNA extraction and transcriptome sequencing were harvested from three plants. Tissues were gently rinsed and subsequently cut into small pieces. All materials were immediately frozen in liquid nitrogen and stored at − 80 °C before use.

### RNA extraction

Total RNA was extracted using the RNA plant Plus Reagent (TianGen, Beijing, China) according to the manufacturer’s protocol. The extracted RNA concentration and integrity were assessed using the RNA Nano 6000 assay kit with the Agilent Bioanalyzer 2100 system (Agilent, CA, USA). For PacBio sequencing, the RNA concentration and integrity were assessed using the Fragment Analyzer system (Agilent, CA, USA). The A260/A280 ratio ranging from 1.9 to 2.0, concentration above 285 ng/μL, and RNA integrity number (RIN) greater than 8.0 were used for subsequent cDNA library construction.

### cDNA library construction and hybrid-sequencing

A total amount of 5 μg RNA was used for cDNA library construction. For Illumina HiSeq sequencing, oligo (dT) beads were used to isolate poly(A)^+^ mRNA. The paired-end libraries were constructed with an insert size of approximately 250 bp. All libraries were sequenced commercially on the Illumina HiSeq 2000 sequencing platform (HiSeq 2000 V3) by the Beijing Genomics Institute (BGI-Shenzhen, China) according to the manufacturer’ protocol to generate paired-end reads of an average length of 150 bp.

For PacBio SMRT sequencing, 1000 ng of mRNA from each tissue was pooled for cDNA library construction. Double-strand cDNA was synthesized according to SMARTer PCR cDNA synthesis kit (Clontech). DNA fragments were selected by BluePippin™ (Sage Science, MA, USA) and ranged over four sizes: 1–2, 2–3, 3–6, and 5–10 kb. DNA fragments after the second large-scale PCR were used as template for SMRTbell library for sequencing. The throughput was about 12 Gb and covered all transcripts in the sample.

HiSeq reads were filtered by discarding the reads with adaptor sequences, reads with more than 5% ambiguous “N” bases, and low-quality reads. The filtered reads were then assembled using Trinity (v2.0.6) with default parameters to generate contigs. These contigs were then processed by sequencing clustering software TGICL (v2.0.6) for redundant Trinity assembled contig removal. Raw PacBio SMRT reads were processed using SMRT analysis server (v2.3) for full-length transcript generation. The obtained transcripts were corrected with filtered HiSeq reads using the LSC error correction tool [69] and subsequently filtered with CD-HIT-EST [70] for the removal of redundant Trinity generated fragments. Finally, the calibrated transcripts were assembled into unique putative transcripts (including contigs and singletons) and unigenes were characterized for subsequent analysis.

### Annotation and differential gene expression analysis

The unigenes were annotated based on sequence similarity using BLASTX against five databases, including non-redundant protein database (Nr), SwissProt, COG, and KEGG protein database. The Pfam annotation for unigenes was done using the HMMER 3.0 package. Sequence description for each unigene was transferred from homologous BLAST hits with E-value< 10^− 5^. GO terms were assigned based on the top BLAST hit using Blast2GO. Genes were obtained by BLASTN using non-redundant nucleotide sequence database (Nt). Functional enrichment of the assigned GO terms was calculated and analyzed by the WEGO software. The distribution of gene functions was illustrated by the GO terms for biological process, cellular component, and molecular function.

Clean reads were mapped to unigenes using Bowtie2 (v2.2.5). The gene expression level was calculated with RSEM (v1.1.12). To compare the difference of gene expression among different samples, the FPKM (Fragments per kilobase of transcript per million mapped reads) method was used for normalization [71]. DESeq2 was used to identify differentially expressed genes (DEGs) (absolute value of log_2_ fold change≥1) after correction of *p*-values (adjusted< 0.05) using the Benjamini-Hochberg procedure (false discovery rate, FDR ≤ 0.001). Highly expressed unigenes characterized from leaf and fruit (high azadirachtin A tissues) libraries were used for candidate mining.

### Analysis of phylogeny and domain architecture of unigenes

The SwissProt database was queried to retrieve all reviewed sequences of alcohol dehydrogenase, CYP450, acyltransferase, and esterase. These sequences were downloaded in FASTA format and aligned with the four kinds of candidates using the ClustalW algorithm with default parameters. Phylogenetic analysis based on multiple alignments of protein sequences was done using the Neighbor Joining [72] method as implemented in MEGA7 and the phylogenetic trees were visualized on iTOL [73]. Accessions of these protein sequences used in phylogenetic analysis are provided in Additional file 2. The protein sequences of the candidates were also searched against the Pfam database in order to get the domain architecture information complementary to that provided by SwissProt.

### Molecular modelling and docking for enzyme-substrate analysis

Modelling of five CYP450s encoded by unigenes was performed using the Phyre2 web portal using the fold recognition method [74]. To characterize the potential active site of binding sites in the protein, we used the web server, 3DLigandSite -Ligand binding site prediction Server [75]. Next, molecular docking was performed with Autodock 4.0 [76] to predict the interactions of four triterpenoids (tirucalla-7,24-dien-3β-ol, azadirone, nimbin, and nimbolide) as substrates for the CYP450 proteins. For covering acting domains present in CYP450 protein, grid spacing was maintained at 0.375 Å. Genetic algorithm (GA) was applied as the searching parameter with 10 GA runs, population size was set to 150. Energy evaluations were set to maximum 25,00,000, considering the maximum number of generations as 27,000. The most favorable docking conditions were in the form of lowest binding energy conformations with H-bonds in cluster. Phymol 2.3 software was used for better analysis of interactions in the protein-ligand complexes obtained from Autodock 4.0 software.

### Validation of hybrid-seq by quantitative real-time PCR and full-length PCR cloning

Ten transcripts were randomly selected to validate their expression from hybrid-seq by quantitative real-time PCR (qRT-PCR). Total RNA was processed with RNase-free-DNase I (TianGen, Beijing, China) following the manufacturer’s instructions, to eliminate potential DNA contamination. First strand cDNA was synthesized using GoScript™ Reverse Transcription System (Promega, Canada). The reactions were performed in triplicate using 2 μL diluted cDNA template in 20 μL total volume. qRT-PCR was performed in 96-well plates on a Bio-Rad CFX96 real-time PCR system (Bio-Rad, CA, USA) using SYBR Green Mix (Bio-Rad, CA, USA). A two-step cycling program was performed, comprising an initial 95 °C polymerase activation for 3 min, followed by 40 cycles of 95 °C for 10 s and 60 °C for 30 s. The melting curve was obtained by heating the amplicon from 65 °C to 95 °C at increments of 0.5 °C per 5 s. The actin gene was used as an internal control to normalize all data. The relative quantitation (ΔΔ*Ct*) method was used to evaluate differences between the tissues for each gene examined. Data analysis was performed using GraphPad Prism version 5 for Windows (GraphPad Software, Inc. La Jolla, CA, USA). The primers for qRT-PCR reactions were listed in Additional file 1.

Ten transcripts were selected for cloning PCR verification, 4 out of 10 (transcript/14449, transcript/14554, transcript/16971, and transcript/16742) were specifically selected as they contained a complete ORF and were significant candidates in the azadirachtin A biosynthetic pathway. The others were randomly selected from the 10 transcripts used in qRT-PCR verification. The cDNA obtained (2 μL) was used in a 50 μL PCR reaction containing 2 μL of forward primer (10 μM), 2 μL of reverse primer (10 μM), and 25 μL of I-5™ 2 × High-Fidelity Master Mix (TsingKe Biotech, Beijing, China). The PCR product was purified using GeneJET PCR Purification Kit (Thermo, USA) and assembled into pJET vector using CloneJET PCR Clone Kit (Thermo, USA). The constructs were then transformed into Trelief 5α chemically competent cells (TsingKe Biotech, Beijing, China) and the amplified constructs were sequenced by Genewiz Company (Genewiz, Beijing, China). The primers for full-length PCR cloning are listed in Additional file 1.

## Supplementary information

**Additional file 1.** Genes primers used for Quantitative Real-Time PCR (qRT-PCR) analysis and full-length PCR cloning.

**Additional file 2.** Details of proteins used for phylogenetic analysis of OSC, ADH, CYP450, ACT, and EST candidates.

**Additional file 3 Table S1.** Concentration of metabolites in leaf, bark, and seed extract of *A. indica.*

**Additional file 4 Table S2.** The expression value of DEGs from transcriptome of *A. indica*.

**Additional file 5 Table S3.** Summary of functional annotations of *A. indica.*

**Additional file 6 Table S4.** KEGG annotation of unigenes from *A. indica.*

**Additional file 7 Table S5.** Pathways and number of unigenes related to secondary metabolites in *A. indica.*

**Additional file 8 Table S6.** Discovery and expression of unigenes involved in terpenoid backbone biosynthesis in *A. indica.*

**Additional file 9 Table S7.** Unigenes involved in the sesquiterpenoid and triterpenoid biosynthesis in *A. indica.*

**Additional file 10 Table S8.** Potential active sites identified on the CYP450s.

**Additional file 11 Table S9.** Details of molecular docking results of 5 CYPs with probable ligands.

**Additional file 12 Table S10.** Expression values of genes upstream of 2,3-oxidosqualene used for analysis in Fig. 6.

**Additional file 13 Table S11.** Molecular docking results of candidate proteins in Fig. 6.

**Additional file 14 Figure S1.** The workflow of gene mining. Letters L, F, R, S, and f represent leaf, fruit, root, stem, and flower, respectively.

**Additional file 15 Figure S2.** Quantitative Real-Time PCR (qRT-PCR) validation of selected unigenes from fruit, leaf, root, stem, and flower tissues of *A. indica*. The relative expression level of each selected gene was determined by the 2^−ΔΔCT^ method. Experiments were conducted in triplicates.

**Additional file 16 Figure S3.** The alignment result of transcript/14449 and AiOSC1. Sequence marked with “*” represents the transcript/14449 from our data.

**Additional file 17 Figure S4.** The amino acid sequence alignment of transcript/16742, AiCYP71BQ5, and MaCYP71BQ5. Same amino acids are shown in white with red background.

## Data Availability

All data generated or analyzed during the current study are included in this article and its supplementary information files. The raw sequencing data have been deposited in the NCBI database under the BioProject ID PRJNA590058 (BioSample: SAMN13316574, SRR12709585, SRR12709584, SRR12709583, SRR12709582, SRR12709581 and SRR10488719). The link accesses to all these data was provided below: https://www.ncbi.nlm.nih.gov/Traces/study/?acc=PRJNA590058.

## Abbreviations

cDNA: complementary DNA
DEGs: Differentially expressed genes
OSC: 2,3-oxidosqualene cyclase
ADH: Alcohol dehydrogenase
CYP450: Cytochrome P450
ACT: Acyltransferase
EST: Esterase
PacBio SMRT: Pacific Biosciences Single Molecule Real Time
FPKM: Fragments per kilobase of transcript per million mapped reads
Nr: Non-redundant protein database
Nt: Nucleotide sequences
COG: Clusters of Orthologous Groups of proteins
KEGG: Kyoto Encyclopedia of Genes and Genomes protein database
GO: Gene Ontology
qRT-PCR: Quantitative real-time
PCR; NGS: Next-generation sequencing
IDI: Isopentenyl-diphosphate δ-isomerase
GGPS: Geranyl diphosphate synthase
FDS: Farnesyl diphosphate synthase
SQLE: Squalene epoxidase
G3P: 3-phosphate glyceraldehyde
MVA: Mevalonate
MEP: Methylerythritol phosphate
IPP: Isopentenyl pyrophosphate
DMAPP: γ-dimethylallyl pyrophosphate
GPP: Geranyl pyrophosphate
FPP: Farnesyl pyrophosphate
DXP: 1-Deoxy-D-xylulose 5-phosphate
MVP: 5-phosphomevalonate
MVPP: (R)-5-Diphosphomevalonate
GGPP: Geranylgeranyl pyrophosphate
HMG-CoA: 3-hydroxy-3-methyl-glutaryl-CoA
PCME: 2-phospho-4-2-C-methyl-D-erythritol
MECP: 2-C-methyl-D-erythritol 2,4-cyclodiphosphate
HMBDP: 1-hydroxy-2-methyl-2-butenyl 4-diphosphate
TMTT: (E,E)-4,8,12-trimethyltrideca-1,3,7,11-tetraene
DXPS: 1-deoxy-D-xylulose-5-phosphate synthase
MDS: 2-C-methyl-D-erythritol 2,4-cyclodiphosphate synthase
HMGR: Hydroxymethyl-glutaryl-CoA reductase
MVK: Mevalonate kinase
PMVK: Phosphomevalonate kinase
GPPS: Geranyl diphosphate synthase
MCS: Myrcene/ocimene synthase
LMS: (R)-limonene synthase
ATNS: (−)-alpha-terpineol synthase
CNS: 1,8-cineole synthase
TM7SF2: Delta (14)-sterol reductase
NSDHL: Sterol-4-alpha-carboxylate 3-dehydrogenase
SMO1: Methylsterol monooxygenase 1
GGPPS: Geranylgeranyl diphosphate synthase
SQS: Squalene synthase
CAS1: Cycloartenol synthase
