# Supplementary figures and images for: Multi-tissue transcriptome analysis using hybrid-sequencing reveals potential genes and biological pathways associated with azadirachtin A biosynthesis in neem (azadirachta indica)
Source: BMC Genomics. 2020 Oct 28;21:749. doi: 10.1186/s12864-020-07124-6 (PMC7592523; doi:10.1186/s12864-020-07124-6)

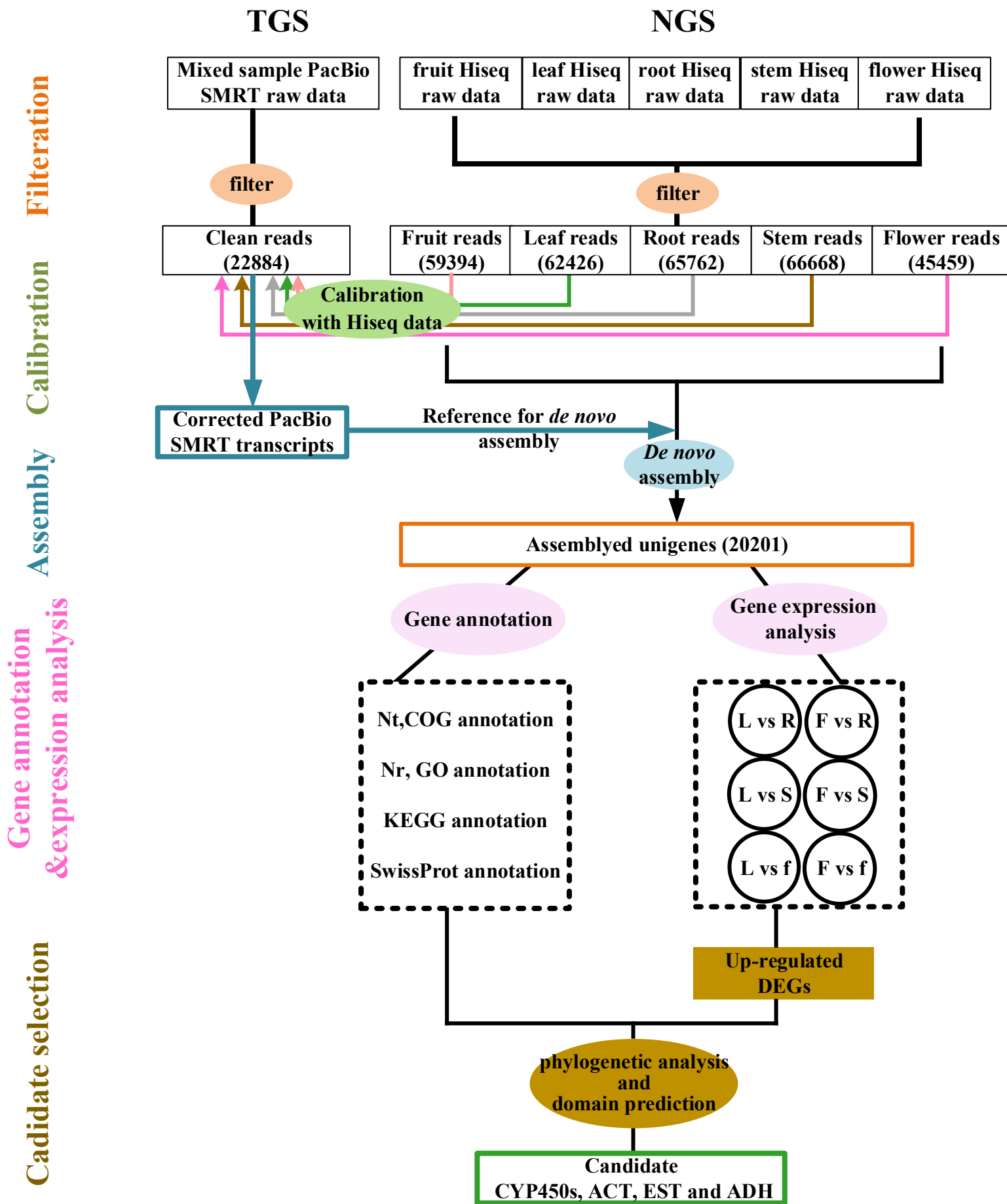

Supplement: Supplementary file 14 — Additional file 14 Figure S1. The workflow of gene mining. Letters L, F, R, S, and f represent leaf, fruit, root, stem, and flower, respectively. [file 12864_2020_7124_MOESM14_ESM.pdf]
