# Supplementary material for: Multi-tissue transcriptome analysis using hybrid-sequencing reveals potential genes and biological pathways associated with azadirachtin A biosynthesis in neem (azadirachta indica)
Source: BMC Genomics. 2020 Oct 28;21:749. doi: 10.1186/s12864-020-07124-6 (PMC7592523; doi:10.1186/s12864-020-07124-6)
Supplement: Supplementary file 15 — Additional file 15 Figure S2. Quantitative Real-Time PCR (qRT-PCR) validation of selected unigenes from fruit, leaf, root, stem, and flower tissues of A. indica. The relative expression level of each selected gene was determined by the 2−ΔΔCT method. Experiments were conducted in triplicates. [file 12864_2020_7124_MOESM15_ESM.pdf]

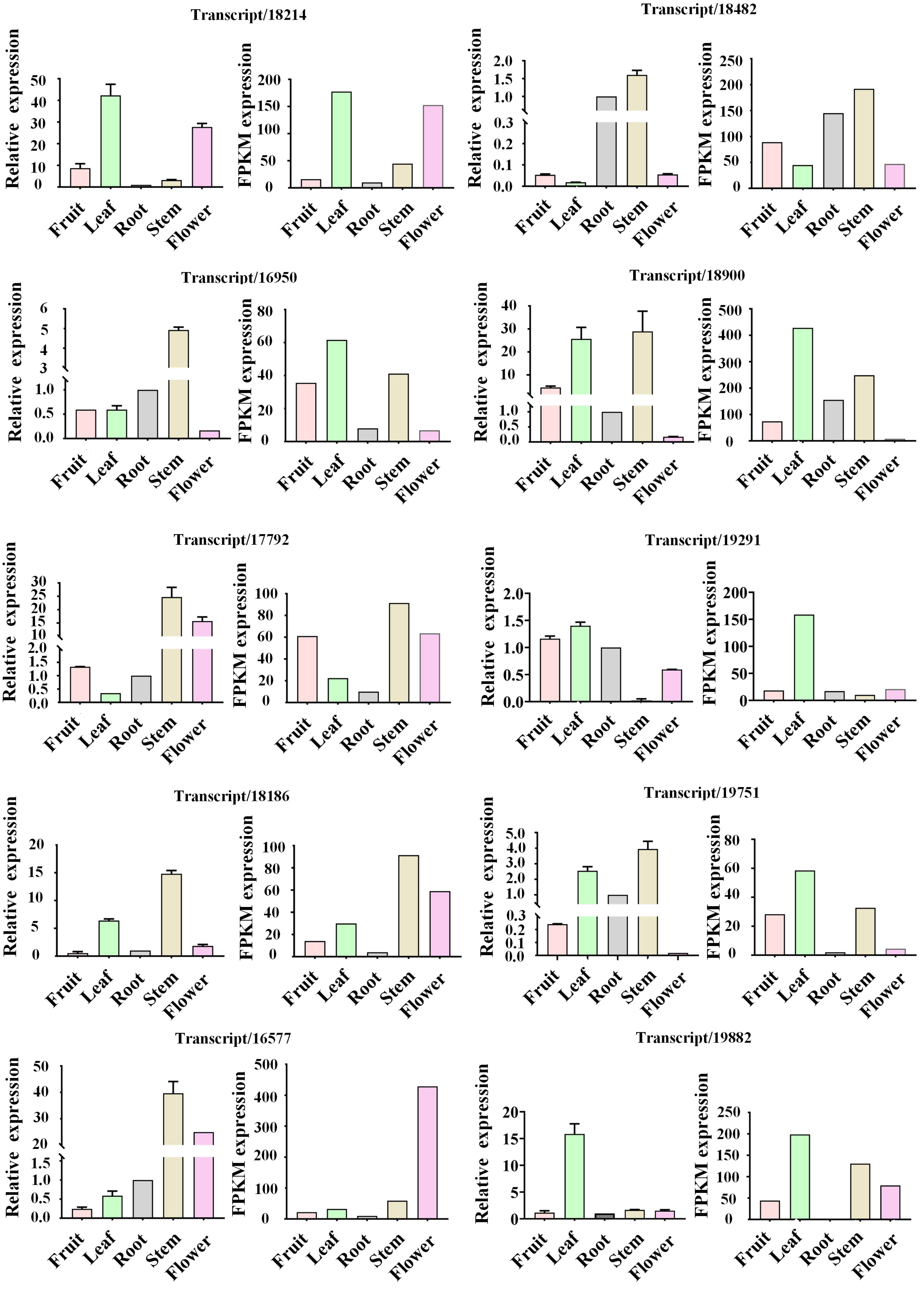
Transcript/18214
Relative expression
50
40
30
20
10
0
FPKM expression
200
150
100
50
0
Fruit
Leaf
Root
Stem
Flower
Transcript/18482
Relative expression
2.0
1.5
1.0
0.3
0.2
0.1
0.0
FPKM expression
250
200
150
100
50
0
Transcript/16950
Relative expression
6
5
4
3
1.0
0.5
0.0
FPKM expression
80
60
40
20
0
Transcript/18900
Relative expression
40
30
20
10
1.0
0.5
0.0
FPKM expression
500
400
300
200
100
0
Transcript/17792
Relative expression
30
25
20
15
10
2.0
1.5
1.0
0.5
0.0
FPKM expression
100
80
60
40
20
0
Transcript/19291
Relative expression
2.0
1.5
1.0
0.5
0.0
FPKM expression
200
150
100
50
0
Transcript/18186
Relative expression
20
15
10
5
0
FPKM expression
100
80
60
40
20
0
Transcript/19751
Relative expression
5.0
4.0
3.0
2.0
1.0
0.3
0.2
0.1
0.0
FPKM expression
80
60
40
20
0
Transcript/16577
Relative expression
50
40
30
20
1.5
1.0
0.5
0.0
FPKM expression
500
400
300
200
100
0
Transcript/19882
Relative expression
20
15
10
5
0
FPKM expression
250
200
150
100
50
0
