# Supplementary material for: Multi-tissue transcriptome analysis using hybrid-sequencing reveals potential genes and biological pathways associated with azadirachtin A biosynthesis in neem (azadirachta indica)
Source: BMC Genomics. 2020 Oct 28;21:749. doi: 10.1186/s12864-020-07124-6 (PMC7592523; doi:10.1186/s12864-020-07124-6)
Supplement: Supplementary file 16 — Additional file 16 Figure S3. The alignment result of transcript/14449 and AiOSC1. Sequence marked with “*” represents the transcript/14449 from our data. [file 12864_2020_7124_MOESM16_ESM.pdf]

```
1*    ATGTGGAAGCTGAAGATTGCAGAGGGTGACAAAAATAGCCCATATATTTCTACAACAAACAATTTCGTTGGAAGGCAAATATGGG
1     ATGTGGAAGCTGAAGATTGCAGAGGGTGACAAAAATAGCCCATATATTTCTACAACAAACAATTTCGTTGGAAGGCAAATATGGG
86*   AATTTGATCCGAACGCCGGAACTGCTGAAGAGCTTGCTGAAGTTGAAGAAGCTCGTCAGAATTTCTACAAGAATCGCCATCAAGT
86    AATTTGATCCGAACGCCGGAACTGCTGAAGAGCTTGCTGAAGTTGAAGAAGCTCGTCAGAATTTCTACAAGAATCGCCATCAAGT
171*  CAAACCTGCTAGTGATCTTATTTTTCGTCTTCAGTTTCTTAGAGAGAAAAACTTCAAGCAAACGATTCCTCAAGTGAAGGTTGAA
171   CAAACCTGCTAGTGATCTTATTTTTCGTCTTCAGTTTCTTAGAGAGAAAAACTTCAAGCAAACGATTCCTCAAGTGAAGGTTGAA
256*  GATGGGGAGGAGATCACATATGACACTGCCACAGCAGCAATGAAGAGGGCTGCTCACTACTTCTCAGCAATTCAGGCTAGCGATG
256   GATGGGGAGGAGATCACATATGACACTGCCACAGCAGCAATGAAGAGGGCTGCTCACTACTTCTCAGCAATTCAGGCTAGCGATG
341*  GCCATTGGCCTGCTGAAAATTCTGGCCCTATGTATTTCCTTCCTCCATTTGTATTCTGCTTGTACATTACAGGACATCTTGATAC
341   GCCATTGGCCTGCTGAAAATTCTGGCCCTATGTATTTCCTTCCTCCATTTGTATTCTGCTTGTACATTACAGGACATCTTGATAC
426*  TGTATTTACAGCTGCTCATCGCAGAGAAGTCCTTCGTTACTTATACAATCATCAGCATGAAGATGGAGGATGGGGAATACACATA
426   TGTATTTACAGCTGCTCATCGCAGAGAAGTCCTTCGTTACTTATACAATCATCAGCATGAAGATGGAGGATGGGGAATACACATA
511*  GAAGCGCCAAGCAGTATGTTTGGTACAGTTTACAGTTATCTTACAATGCGTTTGCTAGGGTTAGGACCCAACGATGGTGAAAACA
511   GAAGCGCCAAGCAGTATGTTTGGTACAGTTTACAGTTATCTTACAATGCGTTTGCTAGGGTTAGGACCCAACGATGGTGAAAACA
596*  ATGCCTGCGCCAGAGCTAGAAAATGGATTCGTGATAATGGTGGTGTCACTTACATTCCCTCTTGGGAAAGAATTGGCTTTCGAT
596   ATGCCTGCGCCAGAGCTAGAAAATGGATTCGTGATAATGGTGGTGTCACTTACATTCCCTCTTGGGAAAGAATTGGCTTTCGAT
681*  TCTTGGTTTGTTTGAATGGGCTGGAACACACCCAATGCCCCCAGAGTTCTGGATGCTTCCTTCTCATTTTCCACTTCATCCAGCC
681   TCTTGGTTTGTTTGAATGGGCTGGAACACACCCAATGCCCCCAGAGTTCTGGATGCTTCCTTCTCATTTTCCACTTCATCCAGCC
766*  CAAATGTGGTGCTTCTGCCGGCTGGTTTACATGCCCTTGTGTTATTTATACGGCAAAAGATTTGTTGGTCCAATCACTCCACTTA
766   CAAATGTGGTGCTTCTGCCGGCTGGTTTACATGCCCTTGTGTTATTTATACGGCAAAAGATTTGTTGGTCCAATCACTCCACTTA
851*  TCAAACAACTGAGAGAAGAACTTCATACAGAGCCTTACGATAAAATCAACTGGAGGAAAGTTCGTCATCAATGTGCAAAGACTGA
851   TCAAACAACTGAGAGAAGAACTTCATACAGAGCCTTACGATAAAATCAACTGGAGGAAAGTTCGTCATCAATGTGCAAAGACTGA
936*  TCTCTACTACCCCCATCCATTCGTACAAGAAGTTCTATGGGATACTCTATACTTTGCTACAGAGCCTCTGCTTACTCGTTGGCCA
936   TCTCTACTACCCCCATCCATTCGTACAAGAAGTTCTATGGGATACTCTATACTTTGCTACAGAGCCTCTGCTTACTCGTTGGCCA
1021* TTGAACAAGTATGTCAGAGAGAAGGCTTTGAAACAAACGATGAAGATCATTCATTATGAAGACCAAAGCAGTCGATATATTACTA
1021  TTGAACAAGTATGTCAGAGAGAAGGCTTTGAAACAAACGATGAAGATCATTCATTATGAAGACCAAAGCAGTCGATATATTACTA
1106* TTGGATGCGTCGAGAAGCCGCTGTGTATGCTTGCTTGTTGGGTGGAGGATCCTGAAGGGGTTGCTTTCAAGAAGCATCTTGAGAG
1106  TTGGATGCGTCGAGAAGCCGCTGTGTATGCTTGCTTGTTGGGTGGAGGATCCTGAAGGGGTTGCTTTCAAGAAGCATCTTGAGAG
1191* AATTGCTGATTTTATTTGGATTGGAGAAGATGGAATGAAAGTTCAGACATTTGGCAGTCAAACATGGGATACTGCTCTTGGACTT
1191  AATTGCTGATTTTATTTGGATTGGAGAAGATGGAATGAAAGTTCAGACATTTGGCAGTCAAACATGGGATACTGCTCTTGGACTT
1276* CAAGCTTTGCTTGCTTGCAATATCGTTGATGAAATTGGACCTGCACTTGCTAAAGGACACGACTACTTGAAGAAAGCTCAGGTGA
1276  CAAGCTTTGCTTGCTTGCAATATCGTTGATGAAATTGGACCTGCACTTGCTAAAGGACACGACTACTTGAAGAAAGCTCAGGTGA
1361* GGGATAATCCAGTGGGTGATTATACAAGCAATTTCCGACACTTTTCCAAAGGAGCATGGACTTTCTCTGATCAAGATCATGGTTG
1361  GGGATAATCCAGTGGGTGATTATACAAGCAATTTCCGACACTTTTCCAAAGGAGCATGGACTTTCTCTGATCAAGATCATGGTTG
1446* GCAAGTTTCAGATTGTACTGCAGAAAGTTTGAAGTGCTGCCTGCATTTCTCAATGCTGCCTCCAGAAATTGTTGGAGAGAAACAT
1446  GCAAGTTTCAGATTGTACTGCAGAAAGTTTGAAGTGCTGCCTGCATTTCTCAATGCTGCCTCCAGAAATTGTTGGAGAGAAACAT
1531* GATCCTGAGAGATTATATGAAGCTGTCAATTTCATACTCTCTCTTCAGGATAAAAATGGTGGAATAGCAGTTTGGGAGAAAGCTG
1531  GATCCTGAGAGATTATATGAAGCTGTCAATTTCATACTCTCTCTTCAGGATAAAAATGGTGGAATAGCAGTTTGGGAGAAAGCTG
1616* GTGCCTCTTTGATGTTAGAGTGGCTCAATCCTGTAGAGTTTCTGGAGGACCTTATTGTTGAGCATACTTACGTGGAATGCACTGC
1616  GTGCCTCTTTGATGTTAGAGTGGCTCAATCCTGTAGAGTTTCTGGAGGACCTTATTGTTGAGCATACTTACGTGGAATGCACTGC
1701* TTCAGCAATCGAGGCATTTGTTATGTTCAAGAAATTATACCCACATCATCGCAAGAAGGAGATTGAAAATTTCCTCGTAAAAGCT
1701  TTCAGCAATCGAGGCATTTGTTATGTTCAAGAAATTATACCCACATCATCGCAAGAAGGAGATTGAAAATTTCCTCGTAAAAGCT
1786* GTACAGTACATTGAAAATGAACAAACTGCTGATGGTTCATGGTATGGAAACTGGGGAGTTTGCTTCTTATATGGAACATGTTTTG
1786  GTACAGTACATTGAAAATGAACAAACTGCTGATGGTTCATGGTATGGAAACTGGGGAGTTTGCTTCTTATATGGAACATGTTTTG
1871* CACTTGGAGGTTTACATGCTGCTGGAAAGACTTACAACAATTGTCTTGCCATTCGTAGAGCAGTTGAGTTTCTGCTTCAAGCACA
1871  CACTTGGAGGTTTACATGCTGCTGGAAAGACTTACAACAATTGTCTTGCCATTCGTAGAGCAGTTGAGTTTCTGCTTCAAGCACA
1956* GAGTGATGATGGTGGTTGGGGAGAGAGCTACAAATCTTGCCCTAGTAAGATATACGTACCTCTTGATGGGAAAAGATCAACTGTG
1956  GAGTGATGATGGTGGTTGGGGAGAGAGCTACAAATCTTGCCCTAGTAAGATATACGTACCTCTTGATGGGAAAAGATCAACTGTG
2041* GTACACACTGCATTGGCTATTCTTGGTTTAATCCATGCTGGGCAGGCTGAAAGAGACCCAACCCCTATTCATCGTGGTGTAAAAT
2041  GTACACACTGCATTGGCTATTCTTGGTTTAATCCATGCTGGGCAGGCTGAAAGAGACCCAACCCCTATTCATCGTGGTGTAAAAT
2126* TGCTGATCAACTCTCAATTGGAGAATGGAGACTTCCCTCAACAGGAAATTATGGGAGTTTTTATGAGAAACTGTATGTTACACTA
2126  TGCTGATCAACTCTCAATTGGAGAATGGAGACTTCCCTCAACAGGAAATTATGGGAGTTTTTATGAGAAACTGTATGTTACACTA
2211* TGCTCAATACAGGAATATTTTTCCTTTGTGGGCTTTAGCTGAATATAGAAGAAAAGTTCCATTGCCTAATTAA
2211  TGCTCAATACAGGAATATTTTTCCTTTGTGGGCTTTAGCTGAATATAGAAGAAAAGTTCCATTGCCTAATTAA
```
