# Supplementary material for: Multi-tissue transcriptome analysis using hybrid-sequencing reveals potential genes and biological pathways associated with azadirachtin A biosynthesis in neem (azadirachta indica)
Source: BMC Genomics. 2020 Oct 28;21:749. doi: 10.1186/s12864-020-07124-6 (PMC7592523; doi:10.1186/s12864-020-07124-6)
Supplement: Supplementary file 17 — Additional file 17 Figure S4. The amino acid sequence alignment of transcript/16742, AiCYP71BQ5, and MaCYP71BQ5. Same amino acids are shown in white with red background. [file 12864_2020_7124_MOESM17_ESM.pdf]

```
                1        10        20        30        40        50
transcrip16742  MTLTMEFRLPSLPVFLSFLLFFLMLVRHWKRSKGQGKPPPGPKPLPILGNL
AiCYP71BQ5      ...................................................
MaCYP71BQ5      ....MEFR...LPVLLSFLLFFLMLVRHWKRSKGQGKPPPGPKPLPILGNL

                        60        70        80        90       100
transcrip16742  HQLADGLPHYAVTKLCRKYGPVMKLKLGQLDAVVVSSPEAAKEVLKTNEIK
AiCYP71BQ5      ...................................................
MaCYP71BQ5      HQLADGLPHYAVTKLCRKYGPVMKLKLGQLDAVVISSPEAAKEVLKTNEIK

                      110       120       130       140       150
transcrip16742  FAQRPEVYAVEIMSYDHSSIVFSPYGDYWREMRKISVLELLSNRRVTSFRS
AiCYP71BQ5      ...............................MRKISVLELLSNRRVTSFRS
MaCYP71BQ5      FAQRPEVYAVEIMSYDHSSIVFSPYGDYWREMRKISVLELLSNRRVTSFRS

                     160       170       180       190       200
transcrip16742  IREDEVWNLVQFISENEGCIINLSERIFTMTNDIISRAAFGNKCDDQHNFT
AiCYP71BQ5      IREDEVWSLVQFISENEGCIINLSERIFTMTNDIISRAAFGNKCDDQHNFT
MaCYP71BQ5      IREDEVWNLVQFISENEGCIVNLSERIFIMTNDIVSRAAFGNKCDDQHNFT

                    210       220       230       240       250
transcrip16742  ALLEEILQIGAGFAIADLYPSLTFLRPLTGMKPALERIHKKMDKILEEIVT
AiCYP71BQ5      ALLEEILQIGAGFAIADLYPSLTFLRPLTGMKPALERIHKKMDKILEEIVT
MaCYP71BQ5      ALLEEILQIGAGFAIADLYPSLTFLRPLTGMKPALERIHKKMDKILEQIVT

                   260       270       280       290       300
transcrip16742  EHQIKRKAAAKNNTEFEEEDLVDTLLNYAEANKNEFHLTTDQVKAVTLDIF
AiCYP71BQ5      EHQIKRKAAAKNNTEFEEEDLVDTLLNYAEANKNEFHLTTDQVKAVTLDIF
MaCYP71BQ5      EHQIKRKAAAKNNTKFEEEDLVDTLLNYAEANKNEFHLTTDQVKAVTLDIF

                  310       320       330       340       350
transcrip16742  SAGSETSATSMEWAMSELLKNPRVMKKAQEEVRQACKGKSKIREADIQNLE
AiCYP71BQ5      SAGSETSATSMEWAMSELLKNPRVMKKAQEEVRQACKGKSKIREADIQNLE
MaCYP71BQ5      SAGSETSATSMEWAMSELLKNPRVMKKAQEEVRQACKGKSKIKETDIQNLE

                 360       370       380       390       400
transcrip16742  YLKLVIKETFRLHAPGPFTPREARETCEIGGYTIPAKAKILINLHAMGRDP
AiCYP71BQ5      YLKLVIKETFRLHAPGPFTPREARETCEIGGYTIPAKAKILINLHAMGRDP
MaCYP71BQ5      YLKLVIKETFRLHAPGPFTPREARETCEIGGYTIPAKAKILINLHAMGRDP

               410       420       430       440       450
transcrip16742  TIWKDPECFQPERFEGSSIDFKGNHFELIPFGGGRRICPGISFATANIELG
AiCYP71BQ5      .IWKDPECFQPERFEGSSIDFKGNHFELIPFGGGRRICPGISFATANIELG
MaCYP71BQ5      TIWKDPECFRPERFEGSSIDFKGNHFELIPFGGGRRICPGISFATANIELG

              460       470       480       490       500
transcrip16742  LAQMMYHFDFKLPNGKSLEDLDMDENFGMTCRRKENLQVIATTRIPFEK
AiCYP71BQ5      LAQMMYHFDFKLPNGKSLEDLDMDENFGMTCRRKENLQVIATTRIPFE.
MaCYP71BQ5      LAQMMYHFDYKLPNGKSLEDLDMNENFGMTCRRKENLQVIATTRIPFQK
```
